# Interleukins expression by rat Lymphocytes exposed to chronic intermittent normobaric hypoxia

**DOI:** 10.3389/fphys.2025.1520174

**Published:** 2025-03-26

**Authors:** Rodrigo Calderon-Jofre, Giuliano Bernal, Daniel Moraga, Fernando A. Moraga

**Affiliations:** ^1^ Laboratorio de Fisiología, Hipoxia y Función Vascular, Departamento de Ciencias Biomédicas, Facultad de Medicina, Universidad Católica del Norte, Coquimbo, Chile; ^2^ Laboratorio de Biología Molecular y Celular del Cáncer, Departamento de Ciencias Biomédicas, Facultad de Medicina, Universidad Católica del Norte, Coquimbo, Chile; ^3^ Departamento de Medicina, Facultad de Ciencias de la Salud, Universidad de Tarapacá, Arica, Chile

**Keywords:** interleukins, lymphocytes, chronic intermittent hypobaric hypoxia, hematological response, inflammation

## Abstract

Acute and chronic hypoxia modulate the expression of inflammatory mediators known as cytokines. However, studies in chronic and intermittent hypobaric or normobaric hypoxia, like those described in miner’s population, are scarce or absent. In this study, we evaluate the effect of chronic intermittent normobaric hypoxia (CINH) on the hematological response and the expression of lymphocyte cytokines IL-1, IL-2, IL-6, and IL-10 in rats. A total of 20 Sprague-Dawley rats were divided into two groups: a) CINH (FiO_2_ 10%, n = 10) and b) Control (normoxic, n = 10). Systolic arterial pressure and heart rate were measured using a tail-cuff sensor. Blood samples were obtained from both groups for hematological studies, and expression of cytokines obtained from lymphocytes was determined by RT-PCR. Hematocrit, hemoglobin, platelet count, and hematological constant were elevated, and leucocyte count decreased in CINH rats. In addition, systolic arterial pressure in CINH rats was significantly increased (over 50%). Cytokine expression from lymphocytes showed that IL-2, and IL-10 increased by 140% and 38%, respectively; IL-6 showed no significant change, while IL-1β expression decreased by 18%. In this regard, CINH could activate an inflammatory response mediated by IL-2. However, this response could be attenuated by increased IL-10 expression, a known anti-inflammatory cytokine, and decreased IL-1β and IL-6 expression, indicative of an adaptation mechanism to CINH.

## Introduction

High altitude exposes people to an environment where factors like hypobaric, cold, low air humidity, and high ultraviolet radiation make adaptation difficult. To date, approximately 23 million people live or work at altitudes between 3,500–5,000 m (Tremblay and Ainslie, 2021), exposing them to an environment with reduced barometric pressure, and hence a reduction in oxygen availability, a condition called hypoxia (Bärtsch and Gibbs, 2007; Parati et al., 2018). Based on the periodicity of exposure, hypoxia can be defined as acute (observed in tourist, climbers, and hikers), chronic (people who live permanently at high altitudes, between 3,000 and 5,000 m), and intermittent (people who alternate exposure to hypoxia and normoxia) (Viscor et al., 2018). However, the term “intermittent hypoxia” can be referred to as Episodic intermittent hypoxia, observed in obstructive sleep apnea (OSA) or chronic intermittent hypobaric hypoxia (CIHH). This condition is observed in people who work a shift system where they work at high altitudes and rest at sea level (Viscor et al., 2018).

The hypoxia-inducible factor (HIF) is a master regulator of the cellular response to low oxygen. HIF is a heterodimeric transcription factor, encompassing a HIF-1α and a HIF-1β subunit. Oxygen sensitivity is accomplished through the action of prolyl hydroxylase domain proteins that hydroxylate proline residues of the HIF-1 subunits, resulting in ubiquitination and proteasome-dependent degradation of HIF-1 when oxygen is present. In response to hypoxia, this prolyl-hydroxylase is inactivated, and HIF-1α dimerizes with HIF-1β and translocate to the cellular nucleus, activating a variety of genes involved in many processes such as glycolysis, angiogenesis, proliferation, migration, autophagy, and apoptosis, amongst others (Semenza, 2020). However, hypoxia conditions also promote an inhibition of mitochondrial oxidative phosphorylation by uncoupling the electron transport chain, causing increased production of reactive oxygen species (ROS) in macrophages, which activates and stabilizes HIF-1α (Semenza, 2020). HIF-1α also increases the formation of neutrophil extracellular traps (Khawaja et al., 2020) and neutrophil survival by inhibiting apoptosis and triggering the nuclear factor NF-kB, which plays a central role in stimulating the release of proinflammatory cytokines such as IL-1β, IL-6 and Tumor Necrosis Factor-alpha (TNFα) (Kiani et al., 2021). In humans, hypoxia induces lymphocytopenia and neutrophilia, causing alterations of specific components of the immune system, such as a reduction in T lymphocyte levels (CD4^+^) and an increase in natural killer (NK) cells (Mazzeo, 2005). Therefore, the key regulators of transcriptional responses in hypoxia and inflammation are HIF and NF-kB, respectively. However, the precise mechanism remains elusive (D’Ignazio et al., 2016).

Early studies have shown that acute exposure to hypobaric hypoxia (4,300 m) for 12 days produces IL-6 overexpression, mediated by β_2_-adrenergic stimulation, and remains elevated for several weeks in response to α-adrenergic activation (Mazzeo et al., 2001). Another study conducted in humans showed that acute exposure to hypoxia induces a significant increase in IL-6, while other proinflammatory cytokines are not altered (Klausen et al., 1997). This cytokine was also increased in subjects who passively ascended to an altitude of 3,500 m, reaching its highest concentration after 30 h of permanence at this altitude (Hartmann et al., 2000).

Studies performed in animal models have shown that chronic hypoxia induces strong expression of proinflammatory cytokines IL-6, IL-1β, and TNFα in immune cells of rats subjected to this stress, and after 28 days, IL-6 levels remain elevated, whereas the latter two returned to normal (Liu et al., 2009).

Nowadays, many of these studies are carried out preferably in conditions of acute and chronic hypoxia. However, few reports describe the effect of chronic and intermittent hypoxia on the expression of cytokines in the lymphocytes of animals subjected to this stress. Therefore, the objective of this study was to determine the effect of chronic and intermittent normobaric hypoxia on lymphocyte expression of IL-1β, IL-2, IL-6 and IL-10 in rats.

## Subjects, materials and methods

### Animals

Twenty young male Sprague-Dawley rats (age: 2.0 months; weight: 250 g, handled in accordance with the Guidelines for Humanitarian Treatment of Laboratory Animals) were used. Two animals per cage were housed and provided with food (10 g/day of pellet per rat) and water using current dispensers. Ambient condition was kept at 22°C along with a 12-h light–dark cycle. All protocols used in the present study were approved by the Ethics Committee of Facultad de Medicina, Universidad Católica del Norte, Coquimbo, Chile (Resolution CEC-FAMED #01/09).

### Hypoxia protocol

Rats were randomly divided into two groups: A Chronic Intermittent Normobaric Hypoxia group (CINH, N = 10), where animals were exposed to 24 h of hypoxia followed by 24 h of normoxia, for 30 days, and a normoxia group (CONTROL, N = 10). The intermittent hypoxia model was performed by reducing the inspired fraction of oxygen (FiO_2_) to 10%, using certified mixtures of oxygen and nitrogen, with a tolerance of ± 0.1% (AGA-Linde Chile). Animals were installed into a sealed chamber (70L*40W*30H, in centimeter), with an input flow of 4–5 L/min. To assure oxygen concentration and an adequate ventilation into the chamber, FiO_2_ was measured with an oxygen sensor (G1690-Greisinger, GHM Messtechnik GmbH) and CO_2_ concentration was measured with an air quality tester (JD-3002, Dongguan Jinlide Electronic Technology Co.). To avoid accumulation of CO_2_ (400–800 ppm) and humidity inside the chamber, we used a CO_2_ absorbent (GE, AMSORB® Plus, Armstrong Medical Limited) and silica gel for humidity. The control group was placed in the same room under the same conditions with the exception of hypoxia.

### Measurements

Body weight (BW, g), systolic blood pressure (SBP, mmHg), and heart rate (HR, bpm) were measured in rats at basal conditions, every 5 days until protocol was completed. BW was measured using a counting balance (Acculab V-1200), SBP and HR were measured using an inflatable tail-cuff and non-invasive pressure sensor (MLT125 R cuff and transducer, Panlab, AdInstruments), as previously described by our laboratory (Lopez et al., 2009; Moraga et al., 2018). The signal was transmitted through a preamplifier to a data-acquisition system (NIBP controller, Model ML126, AdInstruments), and the average value of six consecutive measurements was calculated. All measurements were performed by placing the animals inside a movement-limiting Plexiglas chamber for 10–20 min at a temperature of 22°C.

After 30 days of the protocol, the animals were anesthetized using sodium thiopental (100 mg/kg i.p.), blood samples were taken from the vena cava to be processed immediately, and the animals were euthanized by exsanguination.

### Blood sample processing

#### Hematological parameters

1 mL of venous blood was taken in EDTA tubes for study of hematological parameters, using a hematological counter (ADVIA 60 Hematology System, Siemens). Hemoglobin concentration (g/dL), hematocrit (%), red blood cell count (RBC, cells/L), mean corpuscular volume (MCV, fL), mean corpuscular hemoglobin (MCH, pg), mean corpuscular hemoglobin concentration (MCHC, g/dL), white blood cells (WBC, cells/mm^3^) and platelets (cells/mm^3^) were reported. In addition, 4 mL of venous blood was taken in EDTA tubes for lymphocyte extraction.

#### Lymphocyte purification

Lymphocytes were obtained from 4 mL of whole blood, by centrifugation in a gradient of Ficoll-Paque Plus (Amersham Biosciences) according to the manufacturer’s instructions. Briefly, the blood sample taken with EDTA was diluted 1:1 (vol/vol) in buffered saline solution at pH 7.4. The diluted sample was mixed by gently rotating and subsequently 4 mL of diluted blood was put in a tube with 4 mL Ficoll-Paque Plus (Amersham Biosciences) without mixing. Subsequently, the samples were centrifuged at 400 *g* by 30 min. To improve lymphocyte extraction, the phase between the plasma and the Ficoll-Paque layer was extracted and centrifuged again at 500 g for 10 min and stored until analysis.

#### RNA extraction and quantification

RNA was extracted from purified lymphocytes using the SV Total RNA Isolation kit (Promega, Madison, WI). cDNA was synthesized by reverse transcription using random hexamers and the Superscript First-Strand Synthesis System for RT-PCR kit (Invitrogen Life Technologies). Procedures were carried out according to the manufacturer’s instructions. PCR was performed using the primers indicated in Table 1. The concentration of each RNA was determined by absorbance at 260 nm.

**TABLE 1:** Sequences of primers used.

Target		Sequence (5′ → 3′)	Amplicon size (pb)
IL-1β	Forward	TCTGTGACTCGTGGGATGAT	320
	Reverse	CTTCTTTGGGTATTGTGTGG	
IL-2	Forward	GCGCACCCACTTCAAGCCCT	354
	Reverse	CCACCACAGTTGCTGGCTCA	
IL-6	Forward	TTGACAAGCCACTGCCTTCCC	359
	Reverse	GGTGAGGAGCACGTAGTCGG	
IL-10	Forward	CAATAACTGCACCCACTTCC	350
	Reverse	ATTCTTCACCTGCTCCACTG	
β-actin	Forward	CCGACGGTCAGGTCATC	350
	Reverse	CTCATCGTACTCCTGCTTG	

#### RT-PCR

Assay conditions were established for each pair of primers indicated in Table 1. For each sample, two independent preparations were performed to determine the range of cycles between thresholds at which there was no detectable amplification and up to saturation, where there was no greater increase in amplicon intensity with increasing amplification cycles. PCR amplification was carried out from cDNA synthesized from 1 μg of total RNA, with 1 unit of Taq polymerase (Promega), 10 mM Tris·HCl pH 9.0, 50 mM KCl, 0.1% Triton X-100, 1.5 mM MgCl_2_, 0.2 mM dNTP mix, and 1 μL of each resulting cDNA was used for PCR amplification of IL-1β, IL-2, IL-6, and IL-10. β-actin was used as a housekeeping gene (Table 1). Quantitative real-time PCR assays were performed in a StepOne thermal cycler (Applied Biosystems, CA, United States). The reaction procedure was as follows: initial denaturation step at 95°C for 15 min and 40 cycles (95°C for 30 s; 60°C for 30 s; 72°C for 30 s). The PCR products and molecular weight standard were separated by electrophoresis on 1% agarose gels with ethidium bromide and visualized under UV light. The optical density of the bands of the PCR products were quantified by densitometry using Scion Image Software (Beta 4.02 Win; Scion Image). Relative expression for each gene was expressed as IL-1β, IL-2, IL-6, and IL-10 divided by β-actin expression.

### Statistical analysis

All data were analyzed using Prism 8.4 software (GraphPad Software, San Diego, United States) values are presented as mean ± standard deviation (SD). Changes of body weight, SBP and HR pre and post protocol were analyzed using one-way ANOVA followed by Newman-Keuls *post hoc* test. Differences between Control and CINH in hematological variables and cytokines expression were analyzed with unpaired t-Test. Finally, significant difference for each analysis was considered when *p* < 0.05.

## Results

### Body weight and cardiovascular response

Body weight and cardiovascular variables such as SBP and HR are shown in Table 2. We observed that after 30 days, both groups exhibit a significant increase in BW. However, control rats showed a higher and more significant increase in BW compared to rats exposed to CINH. In addition, animals exposed to CINH showed a significant increase in systolic arterial pressure compared to pre-CINH and post-control, without modifications to heart rate.

**TABLE 2:** Body weight and cardiovascular response to Chronic Intermittent Normobaric Hypoxia.

	Control		CINH	
	Pre	Post	Pre	Post
Number (N)	10	10	10	10
Weight (g)	182 ± 3	228 ± 3*	178 ± 3	208 ± 5*^,†^
SBP (mmHg)	130 ± 6	132 ± 6	134 ± 4	164 ± 10*^,†^
HR (bpm)	205 ± 12	213 ± 12	207 ± 13	205 ± 18

Values reported expressed as mean ± SD. SBP, systolic blood pressure, HR, heart rate. *p < 0.05 (Pre vs. Post); p < 0.05 (Control vs. CINH).

### Hematological parameters

Hematocrit (Hct), erythrocyte, leucocyte and platelet count, and hematological constant are shown in Table 3. All these parameters increased in rats exposed to CINH, except the leucocyte count, which decreased to 11.5 ± 2.6 × 10^3^/mm^3^ compared with 14.3 ± 3.9 × 10^3^/mm^3^ in control animals (p < 0.05).

**TABLE 3:** Hematological response to Chronic Intermittent Normobaric Hypoxia.

	Control	CINH
Number (n)	10	10
RBC (×10^12^ /L)	8.7 ± 0.6	9.2 ± 0.6*
Hemoglobin concentration (g/dL)	14.8 ± 0.7	16.5 ± 0.7*
Hematocrit (%)	45.1 ± 2.7	50.1 ± 3.5*
MCV (fL)	51.8 ± 0.8	54.3 ± 1*
MCH (pg)	17.1 ± 0.5	17.9 ± 0.7*
MCHC (g/dL)	32.9 ± 0.7	32.9 ± 1.1
WBC (×10^3^/mm^3^)	14.3 ± 3.9	11.5 ± 2.6*
Platelets (×10^3^/mm^3^)	388.6 ± 395.9	694.2 ± 344.2*

Values reported expressed as mean ± SD. RBC, red blood cell; MCV, mean corpuscular volume; MCH, mean corpuscular hemoglobin; MCHC, mean corpuscular hemoglobin concentration; WBC, white blood cells. *p < 0.05 (Control vs. CINH).

### Interleukin expression

**FIGURE 1:**
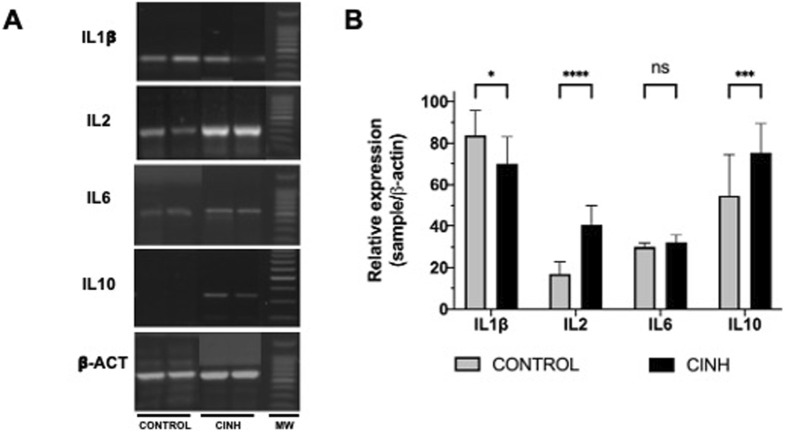
**(A)** RT-PCR products for IL-1β, IL-2 IL-6, IL-10 and β-actin, obtained from lymphocytes in normoxic rats (Control) and exposed to chronic and intermittent normobaric hypoxia (CINH). MW, Molecular Weight. **(B)** Relative change of expression of cytokines. Data expressed as mean ± SD. Open bars, Control; Close bars, CINH. *p < 0.05, ***p < 0.0005 and ****p < 0.0001.

A representative blot of the expression of cytokines is shown in Figure 1A and analysis of expression is observed in Figure 1B. Our results show a significant increase in the IL-2 and IL-10 at 140% and 38%, respectively, in CINH conditions compared to the expression from control lymphocytes (p < 0.05). In contrast, IL-1β decreased its expression by 18% compared with control condition (p < 0.05). However, no significant change in IL-6 expression was observed.

## Discussion

This study provides new insights regarding the expression of interleukins by lymphocytes in rats exposed to CINH.

In our model of CINH, the period of normobaric hypoxia of 10% was maintained for 24 h followed by 24 h in normoxia, for a total of 30 days. This protocol aims to emulate the periods of exposure to which miners are subjected in northern Chile. Previous models using rats exposed to chronic intermittent hypobaric hypoxia (CIHH) for periods of 48 h or chronic hypobaric exposure considered those animals intolerant to hypoxia since they reached hematocrit values of approximately 70%, in addition to loss of body weight and increased vascular resistance, contributing to pulmonary and systemic hypertension (Siqués et al., 2006; Lopez et al., 2009; Moraga et al., 2018). In contrast, our model of CINH demonstrates an increase in the hematological response (nearly 9%) with an increase in body weight, supporting the definition of tolerant animals to hypoxia (Lopez et al., 2009; Moraga et al., 2018), and similar observations were previously described in other models of CIHH (Germack et al., 2002). These data support that changes in blood parameters depend on the duration of exposure to hypoxia, and the increase in hematocrit described in our study (near 9%), can be related to an increase in blood viscosity and therefore explain in part the elevation in systemic blood pressure (Merrill, 1969). However, no change in hematocrit was found in episodic hypoxia associated with very short repetitive exposure in previous model of obstructive sleep apnea in rats (Fletcher et al., 1992). This model has been related with a potentiation in the activity of carotid body, causing an overflow of sympathetic activity with expression of hypertensive mechanisms mediated by proinflammatory cytokines such as IL-1β, IL-6 and TNFα, and this response was abolished by use of anti-inflammatory ibuprofen (Del Rio et al., 2012; Iturriaga et al., 2015; 2022). In addition, the increase in systolic blood pressure observed agrees with results obtained in models of chronic and CIHH exposure (Siqués et al., 2006; Lopez et al., 2009; Moraga et al., 2018; López et al., 2021).

In contrast with the increased number of erythrocytes in response to exposure to CINH, we observed that leucocyte count was decreased by 19.6% in those rats subjected to CINH compared to normoxic rats. This data agrees with previous studies showing leucopenia induced by hypoxia, with a considerable decrease in CD4^+^ T lymphocyte count (Pedersen and Steensberg, 2002; Thake et al., 2004; Mazzeo, 2005). In addition, lymphocytes respond and adapt to hypoxic stimulation by expressing and releasing various cytokines (Facco et al., 2005). One of the cytokines evaluated in this work was IL-1β, which is a proinflammatory molecule with pleiotropic effects on various cells, including an inhibitory effect that determines tumor regression (Dinarello, 1996; Naldini and Carraro, 2005). In the cascade of cytokines related to inflammation, IL-1β together with TNFα are early released from the inflammatory site, which in turn induces the release of IL-6 (another proinflammatory cytokine) and IL-10, which has an anti-inflammatory role, reduces the production of IL-1β and TNFα. In this regard, a study conducted in humans subjected to chronic and intermittent hypoxia showed an increase in IL-6 and IL-10 levels, associated with no changes in systemic pro-inflammatory cytokines TNFα, IL-1β, and IL-8 levels in plasma (Wang et al., 2007), supporting our present findings, where we observed that lymphocytes from rats exposed to CINH have an 18% decrease in IL-1β expression, with an associated increase in IL-10 levels. In contrast, previous reports showed that cultured human mononuclear leukocytes subjected to 40 h of continuous hypoxia dramatically increased IL-1β expression (Naldini et al., 2001). Additionally, a study in Sprague-Dawley rats showed that models of episodic intermittent hypoxia (OSA model, using 15 s of 5% O_2_, followed by 45 s in normoxia, for 3 h daily) induced a marked increase in serum levels of IL-1β and TNFα (Nácher et al., 2009). These differences are probably due to the different intermittency times of hypoxia, which would suggest that interleukin-producing cells adapt to the hypoxic stimulus by reducing the pro-inflammatory stimulus against chronic and intermittent hypoxia, probably through the expression of another systemic anti-inflammatory cytokine such as IL-10.

In addition, IL-10 can inhibit T cell activation, preventing tissue damage mediated by hypoxia. IL-10 is produced primarily by lymphocytes Th2 and Th0 CD4^+^, lymphocytes B, and macrophages (Grilli et al., 2000). The inflammatory reaction triggered by hypoxia requires activation of NF-κB, which in turn induces the expression of chemokines and cytokines. IL-10 induces the attenuation of NF-κB and the consequent decrease of chemical mediators induced by this factor (Miguel-Carrasco et al., 2010). In our work, CINH induced a 38% increase in the expression of IL-10 in lymphocytes of exposed rats, compared to lymphocytes isolated from normoxic rats, which would indicate a protective mechanism against inflammation mediated by other cytokines, inhibiting the expression of IL-6 and decreasing the expression of IL-1β in these cells. Our results agree with those previously reported, where a group of individuals was subjected to 12% O_2_, 1 h daily, 5 days a week, for 8 weeks, after which it was determined that plasma levels of IL-10 increased (Wang et al., 2007).

IL-2 is a 15 kDa cytokine produced almost exclusively by activated T cells and promotes the proliferation of lymphocytes, macrophages, and NK cells. It also participates in the differentiation of CD4^+^ T lymphocytes into Th1 and Th2 effector cells while inhibiting the differentiation of Th17 cells (Hoyer et al., 2008). In our study, we showed that CINH induces a marked increase in this proinflammatory cytokine, which correlates with previous studies, where intermittent exposure to hypoxia (OSA model) stimulates the synthesis of IL-2, IL-8, and TNFα among other cytokines (Naldini and Carraro, 1999; Ryan and McNicholas, 2008). On the other hand, in a study carried out on T lymphocytes, a drastic reduction in the expression of IL-2 was observed when these cells were exposed to 45 min of a pO_2_ < 40 Torr, even after 18 h of recovery in normoxia (Zuckerberg et al., 1994), suggesting again that the cells adapt to different hypoxic conditions. In our model, we speculate that increased IL-2 expression is not related to an increase in lymphocyte proliferation. This is supported by previous studies, where despite inducing the release of IL-2 in lymphocytes exposed to hypoxia during 40 h, IL-2 levels did not reach the necessary concentration to induce cell proliferation, since immune cells subjected to hypoxia express less mitotic cyclins with the consequent inhibition of their growth (Naldini and Carraro, 1999). In addition, unpublished studies performed in rats exposed to CINH of 4,600 m show decreased expression of pro-inflammatory IL-1β and preserved expression of IL-6 measured in lymphocytes, which could be associated with increased anti-inflammatory interleukin-10 levels. In the same line, unpublished data from our group performed in humans supports evidence of an increase in anti-inflammatory IL-10 production with a reduction in the expression of IL-1α and IL-1β, a low expression of IL-2, IL-6 and TNFα in acclimatized workers exposed to CIHH at 4,500 m respect of a similar population working at sea level. Nevertheless, the reduced number of cytokines studied, and the lack of information regarding plasmatic levels would be consider important limitations of the present study that must be considered in future studies, as well as expression of different transcription factors related to inflammatory pathways and their possible contribution in response to CIHH. In this sense, work in progress from our group are focused on study the expression of cytokines and transcription factors related to inflammatory response mediated by T lymphocytes in a similar animal model of CINH presented here.

In conclusion, the results presented in this work show that lymphocytes of rats exposed to CINH activate an inflammatory response mediated by IL-2. However, this response tends to be attenuated since these cells also increase IL-10 expression and hence inhibit IL-6 expression and decrease IL-1β expression, indicating an adaptative mechanism to hypoxia. This suggests that prolonged hypobaric exposure results in the sequential induction of differentially mediated anti-inflammatory activity, most likely due to the contemporary repression of the pro-inflammatory role associated to NF-κB, TGFβ and TNFα. Future studies focused on other cytokines and transcription factors related to inflammatory response are required to elucidate the pathways involved and the long-term implications of these process in people exposed to CIHH.

## Data Availability

The raw data supporting the conclusions of this article will be made available by the authors, without undue reservation.
